# Account of Several Cases of Carditis: With Remarks

**Published:** 1835-07-01

**Authors:** William Stroud

**Affiliations:** Physician to the Northern Dispensary, &c.


*Account of several Cases of Carditis: with Remarks.* Communicated to the Harveian Society, by William Stroud, m.d. Physician to the Northern Dispensary, &c.

(Continued from Vol. III. page 447.)

*Account of a Case of Chronic Carditis, accompanied with partial Pneumonia, and with Hepatitis.*

May 14, 1831. I visited for the first time, as a patient of the Northern Dispensary, William D****r, fifty-three years of age, who has for more than three years laboured under a pectoral complaint, which is aggravated in winter, and somewhat relieved during the summer. The principal present symptoms are pain at the pit of the stomach, and in the arms, with dry cough, confined bowels, disturbed sleep, and occasional headach. During eleven years he followed a seafaring life, but relinquished it about sixteen years since, afterwards worked as a blacksmith, and, finally, as a shoemaker. His habits were formerly intemperate, and five years since the left side of his chest was inflamed.

*Prescriptions.* Aquæ puræ, f℥vj.; Potass. Nitrat. ʒj.; Pulv. Acaciæ gummi, ʒiij.; Tinct. Digital. fʒss.; Tinct. Scillæ, fʒj.; Tinct. Hyoscyam. fʒiss. Sumatur f℥j. ter quotidie.—Extr. Hyoscyam. gr. v. omni vesp. horâ somni.—Extract: Aloes, Extract. Jalap. *aa* gr. xv.; Pulv. Cambog. gr. v.; Pulv. Colocynth. gr. x.; Ol. Croton. gutt. v.; Ol. Juniperi, q.s. fiant pil. xij. quarum sumantur ij. primo mane, prout opus fuerit.—Emplastr. Cantharid. Unguent. Cetacei, *aa* ℥iv.; Antimon. Tart. ʒj.; Ol. Camphor. fʒij.; Fiat ceratum inter scapulas illinendum.

20. He is no better. The pain of his arms extends to his fingers' ends. His cough is troublesome, and his sleep much disturbed.

*Prescriptions.* Præcord. affig^r hirud. vj.—Pulv. Digital. gr. vj.; Pulv. Opii, Pulv. Scillæ, *aa* gr. xij.; Pulv. Glycyrrhiz. ℈j.; Syrupi q. s. Fiant pil. xij. quarum sumatur una omni vesp. horâ somni.—Rep. med. præter Extr. Hyoscyam.

21. He is rather worse, complaining of pain in the pit of the stomach, with nausea, and, when lying on the left side, with pain in the heart. His cough is chiefly troublesome in the morning, and is accompanied by an expectoration slightly tinged with blood. His bowels are inactive, and his urine is scanty. On examination with the stethoscope, the respiratory murmur is tolerably audible, but the sound of the heart's action is dull, while the pulse at the wrist is strong.

*Prescriptions.* Extrah^r per V.S. sang. f℥xij.—Pect. infer. admov^r Empl. Canth.—Aquæ puræ, f℥ivss.; Mucilag. Tragacanth. fʒiss.; Tinct. Digital. fʒss.; Vin. Colchici, fʒij.; Liq. Antimon. Tart., Tinct. Opii Camph. *aa* fʒiv. Sumatur f℥j. ter quotidie.—

Extr. Hyoscyam. gr. v. omni vesp. horâ somni.—Rep. Pil. Cathart. prout opus fuerit.

24. The bleeding gave some relief. The serum of the blood drawn was of a greenish colour. The blister was not applied. His urine is scanty and acrid; and his hands and feet are œdematous. He is drowsy, but the cough disturbs his sleep, and prevents his lying down.

*Prescriptions.* Extr. Hyoscyam. ℈ij.; Elaterii, gr. iv.; Syrupi, q. s. Fiant pil. xii. quarum sumatur una, omni mane et vespere. —Rep. med. præter Pil. cathart. Pect. infer. admov^r Empl. Canth.

26. The blister applied to the lower part of the chest operated well, and relieved the pain of the heart. The elaterium pills produced a considerable discharge of watery liquid, both from the bowels, and from the kidneys; but the legs are, nevertheless, œdematous. He is chilly, weak, and drowsy, with pain in the shoulders, and much shortness of breath. His cough, which is now attended with a copious white expectoration, is chiefly troublesome at night. His sleep is restless, he is unable to assume the recumbent posture, and sometimes wakes in a fright.

*Prescriptions.* Extr. Hyoscyam. ℈ij.; Pulv. Cambog. gr. xij.; Elaterii, gr. iv.; Syrupi, q. s. Fiant pil. xij. quarum sumatur una, omni mane, et repet^r p. o. f.—Mist. Camph. f℥vj.; Potass. Acetat. ʒij.; Tinct. Scillæ, fʒiss.; Tinct. Opii Camph. fʒiv. Sumatur f℥j. ter quotidie.—Pil. Sapon. cum Opio, gr. v. omni vesp. horâ somni.

31. The patient gradually declined, and expired early this morning. His death was probably accelerated by an imprudent removal to a lodging in a neighbouring street, in consequence of an objection which had been made to the low, and damp situation of the kitchen, where he previously lived.

*Post-mortem Appearances.* An inspection having been obtained the next day, the following appearances were observed.

*General Conditions.* The body was robust and well formed, exhibiting little œdema, and no fat. The skin was firm, and the muscles were thick and red. The head and spine were not examined.

*Thorax.* The cartilages of the ribs were nearly ossified, especially those of the sixth pair. The mediastinum was broad, and its cellular membrane dense. The right pleural sac contained a considerable quantity of reddish serum; and owing, perhaps, to the weight of this liquid, and of the diseased liver, descended deeper into the abdomen than its fellow, contrary to the usual occurrence. The inner surfaces of the left pleural sac were universally united by short and firm adhesions, apparently of old standing. The right lung was somewhat inflated, or emphysematous, but otherwise healthy. The left lung was much hepatized, more especially the central parts, which were dense and red, while those near the surface were tolerably sound and spongy. The distinction was very perceptible on pressing the several portions between the fingers, when the one was found crepitous, and the other fleshy. There was no tubercle, vomica, or extravasation, either in the parenchyma of the lungs, or in the bronchial tubes; but the bronchial membrane, which was of a deep red colour, was coated with a little frothy cream-like mucus, and the mesochondriac fibres were very distinct.

The pericardium, with its contents, was of nearly double the usual size. The membrane itself was rather thickened, and contained several ounces of pellucid watery liquid. The substance of the heart was, in general, firm, thick, and red, evidently from the influence of chronic inflammation. The cavities on both sides contained a good deal of soft, red, and broken coagulum. The right auricle was much dilated. Its musculi pectinati were extensive, but loose, exhibiting the appearance of strong fleshy columns, with wide interstices. The right ventricle presented nothing remarkable. The left auricle was dilated, and somewhat thickened. Its auricula propria was singularly contracted, and convoluted in a spiral form, like that of a cornu Ammonis, but quite pervious to the apex. The walls of the left ventricle were very thick. The aorta was much diseased, and, to some distance from the heart, was of nearly double its ordinary size and substance. On slitting it up, the lining membrane was found hypertrophied, presenting a scabrous and irregular surface, roughened by decussating fibres, with intervening indentations and depressions. The aortic valves were somewhat thick and rigid, but less so than the opposite lining membrane, which was slightly ossified. All the other valves, both cardiac and arterial, were sound. The orifices of the coronary arteries were contracted, and not easily discoverable, being concealed by a thin transverse fold, resembling an irregular valve.

*Abdomen.* The peritoneal sac contained a moderate quantity of reddish serum. The omentum was voluminous, and extensively spread over the surface of the intestines, but free from disease, and almost destitute of fat. The stomach was inflated, and of large size. Its lining membrane was of a dark colour. The intestines were generally healthy, but some portions of the ileum were red and contracted. The colon at the sigmoid flexure was abruptly and considerably dilated, but, with this exception, the large intestine was throughout much contracted and indented, containing only a small portion of thin yellow fæces. The liver was, apparently, of nearly double its usual size and weight. On making incisions into its substance, it was found to be universally dense, and uniformly mottled with red and white spots, like some varieties of porphyry. The gall-bladder was small, and inclosed a little dark-coloured and ink-like bile. The spleen, and the kidneys were firm and sound; as were likewise the mesentery, and the urinary bladder, which was empty and collapsed.

Remarks. Several of the observations annexed to the cases previously related, being equally applicable to the present one, need not here be repeated. In a middle-aged man of robust frame, and vigorous constitution, a life of labour, hardship, and intemperance, occasioned chronic inflammation of the heart, liver, and left lung, attended with excessive growth, and increased redness and firmness of texture, but without any peculiar disorganization, or adventitious deposit. The pericardium, which was thickened, contained several ounces of pale serum, but was free from fibrinous exudation. All the cardiac pouches had acquired an increase of muscular structure, which predominated, as usual, in the left ventricle, while the auricles alone were dilated.

This conjunction of hypertrophy with dilatation, which existed not only in the heart, but, also, in the aorta to some distance from it, may serve to show, in opposition to the doctrine of certain eminent authors, that inflammation depends not on mere debility in the part affected, but on a specific irritation, accompanied with various kinds and degrees of functional derangement. The extensive developement of the musculi pectinati, and the remarkable convolution of the left auricula propria, owing, apparently, to a permanent contraction of its spiral muscular fibres, sufficiently prove that the expansion of their cavities was not the result of a simple deficiency of power. The increased and irregular growth of the lining membrane of the aorta had somewhat affected its valves, producing a tendency to ossification, and a partial obstruction of the orifices of the coronary arteries, which, probably, contributed to diminish the violence, and to retard the progress of the disease. This morbid state of the aorta is a frequent consequence of habitual intemperance, especially, perhaps, of indulgence in ardent spirits, and is often the primary cause of cardiac disease, which in this case seems, however, to have been chiefly independent.

When carditis is productive of partial pneumonia, the left lung is, from its vicinity, the part most liable to suffer; and on the same vicinity, apparently, depended, in the present instance, the hepatized state of its central lobules, while those near the surface were comparatively healthy. This occurrence, which is not very common, shows that inflammation does not always attack the whole organ at once; but, in some cases, is successively propagated from one lobule to another. The inflammation which obliterated the cavity of the left pleural sac could not, however, have been produced by this continuous process, and was probably coeval with that of the heart.

Under these circumstances, and in the absence of internal œdema, the left lung was somewhat compressed and contracted, while the right lung, being comparatively unaffected, had become emphysematous, and its pleural sac dilated, so as to contain at length a large quantity of red serum. This effusion was attributable, not to any morbid state of the membrane itself, but to the final obstruction of the circulation, a little before death, by the gradual and unequal failure of vital power; owing to which, also, the blood retained in the heart was in a state of soft and loose coagulation. The inflammation of the bronchia, which was probably secondary to that of the heart and lungs, had not promoted suffocation by any considerable effusion of mucus. The thickened and conspicuous state of their contractile fibres, in this and similar cases, seems to indicate, agreeably to the opinion of some of the older medical writers, that they perform an important office, both in the healthy, and in the morbid conditions of the lungs.

The dropsical state usually attendant on such complaints, had not proceeded to any extent, either in the abdomen, or in the common cellular texture, having been chiefly confined to the serous membranes near the seat of the disease, namely the pericardium, and the right pleural sac. Its limited amount might be ascribed to the early failure of vital power, and to the original soundness of the constitution, to which, also, in conjunction with the hypertrophy of the heart, it was probably owing that the solid viscera of the abdomen were generally firm, and the liver, in particular, was much enlarged and condensed, exhibiting on section a mottled texture, like that of porphyry, the joint result, perhaps, of intemperance, and of cardiac disease. The increased development of the stomach, the dark colour of its lining membrane, and the redness and constriction of some of the intestines, were, no doubt, partly dependent on the obstruction of their circulation, occasioned by the morbid state of the liver and heart.

The symptoms of this case were so obvious and intelligible as to require but little comment. Independently of the evidence furnished by the history of the patient, the affection of the heart was sufficiently denoted by pain in the præcordia, when he lay on the left side; by pain in the epigastrium, with nausea, from irritation of stomach; and by the usual neuralgic pains in the arms, sometimes extending to the fingers' ends. The respiratory murmur was tolerably audible even on the diseased side, in consequence of the superficial lobules being permeable to air, although the central ones were hepatized. The dull sound of the heart's action, in conjunction with a strong pulse at the wrist, was characteristic of hypertrophy. In accordance with the state of visceral disorder, the abdominal secretions were diminished, or vitiated. The bowels were usually confined, the urine was scanty and acrid, the serum of the blood drawn was greenish, and the small quantity of bile found in the gall-bladder was of an inky colour; but, with this exception, neither the pulmonary nor the abdominal symptoms were very conspicuous. Towards the fatal termination, the progress of serous effusion within the chest, and of consequent cerebral congestion, was indicated by difficulty in lying down, and by drowsiness and torpor during the day, while the sleep at night was restless, and sometimes suddenly interrupted with alarm.

In such a state, even slight causes of disturbance, and, more especially, the removal of the patient to another place, should be carefully avoided, since they are very capable, as was suspected in the present instance, of accelerating death. On the other hand, the fatal conclusion may often be retarded, and the symptoms mitigated, by a moderately antiphlogistic regimen, and by occasional small bleedings, blisters to the chest, diuretic medicines, and minute doses of mercury. Owing to its active hydragogue powers, elaterium will sometimes rapidly diminish the internal dropsical effusion, and thus afford a very remarkable, although, of course, merely temporary relief.

*Account of a Case of Chronic Carditis, terminating in Hypertrophy, accidentally discovered after Death.*

April 30, 1833. Mary V*******n, a young woman about twenty-two years of age, was visited, at six o'clock in the evening, by Mr. Williams, of Gray's Inn Lane, who found her in a deplorable state of want and misery, and far advanced in labour. She seemed to be extremely weak and exhausted, and, within half an hour from Mr. Williams's arrival, was safely delivered of a still-born male child, at the full period. She complained much of thirst, and expressed a wish for ale, which was allowed her. Her countenance was pale, and her pulse very small and quick. On the following morning, she was found rapidly sinking, with a pulse scarcely perceptible, and in a state of great anxiety, but perfectly rational and collected. Every suitable attention was paid to her in the course of the day; but, in spite of all the means employed, she died during the ensuing night, about thirty-six hours after delivery. It was ascertained, on inquiry, that she was unmarried, and that, under the guidance of her mother, a woman of violent passions, and grossly addicted to intoxication, she had led an irregular and intemperate life, and had been abroad during the whole of the day preceding her labour, for which no preparations were made. From a very early age she had been subject to pains in the region of the heart, attended with palpitation and with a sense of weight in the chest; as likewise to rheumatic pains of the arms and shoulders.

*Post-mortem Appearances.* An inspection, at which I was present, was made about noon, May 3, by Mr. Williams, and Mr. Edward White, when the following appearances were noticed.

*General Conditions.* The body was free from discolouration, and rather slender, but well formed, and not emaciated. It contained little blood, except in the heart and large vessels, and scarcely any fat. The countenance was natural. The limbs were moderately flexible. The muscles were fleshy, and of a deep red colour. The internal parts exhaled a slightly acid odour, but putrescence had not commenced. The head and spine were not examined.

*Thorax.* The right pleural sac contained more than a pint of red serum. That on the left side was nearly filled up by numerous bands of adhesion, extending all round from the sternum to the spine. The lungs were sound, and supernatant in water. The upper lobes were somewhat emphysematous, the lower and posterior portions were slightly congested and œdematous. In both lungs, the bronchia contained a little frothy mucus, their lining membrane was deeply reddened, and their contractile fibres were very strong and conspicuous. The left lung was rather small. Several of its bronchial tubes were enlarged, and their lining membrane was thickened, so as to exhibit on section white circular patches, with a central perforation.

The heart was unexpectedly found to be the principal seat of disease, being at once dilated and hypertrophied, and nearly of three times its natural size. The two layers of the pericardium, universally and firmly adherent, were fleshy, rough, and of a deep red colour; and, at one spot on the upper part of the right ventricle more than an inch in diameter, was a thick cartilaginous deposit verging to ossification. The inferior vena cava was very large and turgid, but all the other vascular trunks seemed to have been compressed and reduced in size, by the contractile force of the inflamed pericardium; whilst, in consequence of the enlargement of the entire organ, their position was, at the same time, somewhat altered. All the cavities of the heart were to a considerable extent thickened and dilated, and they all contained much blood, chiefly in the state of soft, loose, dark red coagulum, intermixed with some portions which were paler, and of a firmer consistence. The lining membrane of the auricles, especially that of the left, was white and opaque, as if from an interstitial deposit of albumen. The left ventricle, which was the principal seat of hypertrophy, preserved its expanded form and dimensions when cut open, and its fleshy columns were very prominent and distinct.

The cardiac valves, on both sides, but particularly the mitral, were somewhat thickened and opaque, from incipient interstitial deposit, tending in some points to ossification. The aortic valves were in a similar state, but not to the extent of interrupting their function.

The lining membrane of the aorta was free from disease, and the valves of the pulmonary artery were in their usual sound condition.

*Abdomen.* The stomach was large and thin, without rugæ, and contained more than a pint of alimentary liquid exhaling an acid odour. At the lower end of its greater curvature, the lining membrane was reddened by partial inflammation, and the neighbouring veins were enlarged. The intestines, also, were generally red and vascular, as if from irritation or congestion, but were otherwise healthy. The liver was larger than usual, especially the right lobe, the upper surface of which presented a rounded protuberance, elevating the superincumbent diaphragm. The left lobe was rather small and thin. It seemed that, in consequence of the impeded return of its blood, the liver had been generally gorged and hypertrophied. On section, its structure appeared slightly mottled throughout, and a good deal of reddish serum exuded. The gall-bladder was of full size, and contained about two ounces of thin brownish bile, without concretions.

The kidneys were of a deep red colour, and of fleshy consistence; but, together with the spleen, were quite sound. The uterus was in the state wherein it is usually found shortly after delivery, namely, about the size of three fists; its peritoneal coat being whitish, its walls thick and pale, its cervix soft and red, and its internal surface roughened by a little adherent coagulum.

REMARKS. The principal remark suggested by this interesting case of cardiac disease, is the singularity of an active and dissolute life having been compatible with so serious a complaint, which had, apparently, existed from an early age, and was, no doubt, much aggravated by habits of intemperance and irregularity. Independently of deficient nourishment, and mental emotion, death seems to have been at length occasioned by the rapid exhaustion which attended the act of parturition, leaving the diseased heart in a state of inability to maintain an efficient circulation. The failure of vital power was indicated by the copious effusion of bloody serum into the right pleural sac, and by the soft, loose, dark red coagulum found in all the cardiac cavities. The similarity, to a great extent, of the morbid appearances noticed in this case to those in the preceding one, will be obvious on inspection, and precludes the necessity of lengthened observations. The chronic inflammation of the heart had been universal, producing increased thickness and firmness of the pericardium, the muscular fibres, and the lining membrane. The contractile force of the adherent pericardium, not inferior to that of the inflamed pleura, seemed to have compressed and diminished all the vascular trunks except the inferior vena cava, which was beyond its reach; and may, perhaps, by this means have contributed to the dilatation of the cardiac pouches, which were, at the same time, greatly hypertrophied.

*Account of a Case of Chronic Carditis, terminating in Hypertrophy.*

March 11, 1835. Edwin John B*****t, a boy rather less than eleven years of age, and evidently in a very weak and broken state of health, was brought by his mother to my house, as a patient of the Northern Dispensary. About four years since, he is said to have been the subject of severe continued fever, and, during his convalescence, used to wander about the streets, and to sit down on the steps of doorways. His mother believes that, in consequence of this exposure, his feet were repeatedly chilled, and that a foundation was thereby laid for his present complaint, which, with occasional aggravations, marked by rigors, fever, and headach, has ever since been progressively increasing. He has much palpitation of the heart, with hurried breathing, and a dry cough, chiefly troublesome when he first lies down in bed. He lies ill on the right side; and, owing in part to the disturbed and panting state of his respiration, his sleep is interrupted, and attended with feverishness during the night, and with profuse perspiration in the morning. His pulse, in the sitting posture, is 130, and weak; his tongue rather white. He has little thirst or appetite, and formerly used occasionally to vomit his food. His bowels are relaxed, the dejections thin and dark coloured, and the urine scanty, red, and turbid. He has a propensity to pick his nose, which sometimes bleeds, as likewise other parts of his body; is gradually becoming weaker and more emaciated, and since the last week his legs have been œdematous. On close examination, his chest is found to be somewhat unequal and misshaped; its lower region, especially on the right side, yields a feeble respiratory sound, and little resonance on percussion. In the upper part of the chest, and in the back, the respiratory murmur is sufficiently audible. The action of the heart is too powerful, and too widely diffused, being heard in the epigastrium and between the shoulders, and strongly repelling the ear when applied to the left nipple, but is unattended with any peculiar morbid sound. The abdomen, which is prominent, and rather solid, but not hard, bears pressure well. Its lower region is more flatulent, and its upper portion, particularly the right hypochondrium, more firm and tumid, as well as somewhat painful. In conformity with these indications, the case was regarded as hypertrophy of the heart, accompanied with its usual effects, congestion of the lungs, and, probably, enlargement of the liver. The prognosis was, of course, unfavourable, although, on account of the youth of the patient, and his freedom from other disease, it was supposed that an attempt to diminish the action of the heart, and to reduce its bulk, might be made with some prospect of success.

*Prescriptions.* Præcordiis affigantur hirud. vj.—Inter scapulas admovr. Empl. Cantharid.—Aquæ puræ, f℥vj.; Acid. Tartarici, ʒj.; Tinct. Digital. fʒss.; Tinct. Hyoscyam. fʒjss. fiat potio.—Aquæ puræ, f℥vj.; Sodæ Carbonat. ʒj.; Vin. Colchici, fʒij. fiat potio. Sumatur ex alterutrâ dum mixtæ effervescant, f℥j. ter quotidie.—Hydrarg. cum Cretâ, ℈j.; Pulv. Ipecacuan. gr. xv.; Pulv. Digital. gr. iv. Fiant chart. æqual. vj. quarum sumatur una, omni vesp. horâ somni.

17. The leeches extracted a little dark-coloured blood. The blister operated well. The bowels are moderately open, the dejections firm, and the urine more natural. The boy slept well last night, but his sleep was previously disturbed by a dry cough. He perspires much early in the morning, chiefly about the head, and is somnolent during the day. He is rather less disposed to pick himself than before, and his legs are less œdematous. The heart palpitates much, but is free from pain.

*Prescriptions.* Extrahr. per V.S. sang. f℥v. vel vj. Rep. med. antea præscripta.

23. The bleeding was well borne. The blood drawn was thin and dark coloured, with little crassamentum, and much yellow serum. The alvine evacuations are natural. About two pints of red and turbid urine are passed daily. A little coagulated blood is discharged from the nostrils. The sleep is unquiet, and attended with moans and grinding of the teeth. The mind is tolerably cheerful. The body is feeble and wasted. The cough and palpitation are abated. The œdema of the ankles is nearly removed; but the knees, more especially the left knee, are swollen, stiff, and painful; and there is to-day, in the region of the liver, a pain so acute as to occasion cries.

*Prescriptions.* Aquæ puræ, f℥vss.; Potass. Nitrat. ℈ij.; Liq. Potass., Tinct. Iodini, aa fʒss.; Tinct. Hyoscyam., Vin. Colchici, aa fʒij. Sumatur f℥j. ter quotidie.—Pulv. Glycirrhir. gr. xij.; Acetat. Morphiæ, gr. ij.; Syrupi, q.s. Fiant pilulæ vj. quarum sumatur una, omni vesp. horâ somni.

27. The pain above noticed in the region of the liver shifted the same day to the right foot, where it has since continued. The cough is troublesome, and excites pain in the abdomen. The stomach is oppressed on taking food or drink. The bowels are rather confined.

*Prescriptions.* Præcordiis affigr. hirud. vj.—Pulv. Rhei, gr. v.; Hydrarg. Submur. gr. j. Sumantur primo mane, prout opus fuerit. —Aquæ puræ, f℥vss.; Potass. Nitrat. ℈ij.; Tinct. Digital. m. xx.; Tinct. Iodini, Liquor. Potass. aa fʒss.; Vin. Ipecacuan., Tinct. Hyoscyam. aa fʒij. Sumatur f℥j. ter, vel quater quotidie.—Pulv. Ipecacuan., Pulv. Glycyrrhiz. aa gr. vj.; Acetat. Morphiæ, gr. iij.; Syrupi, q.s. Fiant pilulæ vj. quarum sumatur una, omni vespere horâ somni.

31. The leeches drew a sufficient quantity of blood, and occasioned some debility. Where the pain and palpitation had been the greatest, the blood drawn was extremely black. The cough is less frequent, but stronger, and accompanied with a thick and white expectoration; and there is much discharge from the nostrils. The right leg is swollen. He sleeps more in the morning, with much perspiration from the head.

*Prescriptions.* Inter scapulam sinistram et spinam dorsi verticali tractu inseratur setaceum.—Rep. med. sed fiant pil. narcot. cum Acet. Morphiæ, gr. ij. tantum.

April 5. The seton has not yet produced any discharge. The boy passes about a pint of red turbid urine daily, and in general has three dejections, which are rather solid. He is free from pain, and sleeps better, but is otherwise little changed. The cough is occasionally troublesome, and remarkably so when the mind is agitated or depressed. The left temporal artery is often turgid. The swelling of the right leg has nearly subsided.

*Prescriptions.* Curentur ulcera ex setaceo cataplasmate lini.—Aquæ puræ, f℥v.; Sulphat. Magnes. ℥j.; Tinct. Digital., Tinct. Iodini, aa fʒss.; Vin. Colchici, fʒij.; Liquor. Antimon. Tart. fʒiv. Sumatur f℥j. ter quotidie.—Pulv. Ipecacuan., Pulv. Digit., Pulv. Glycyrrhiz. aa gr. vj.; Acetat. Morphiæ, gr. ij.; Syrupi, q. s. Fiant pilulæ vj. quarum sumatur una, omni vesp. horâ somni. —Repetr. primo mane p. o. f. Pulv. Rhei cum Hydrarg. Submur.

10. The seton excites little discharge. The boy grows thinner and weaker, perspires much, has a strong appetite, and little thirst. His bowels are moderately open, and his urine is sufficiently copious. The cough is somewhat abated, but the nostrils discharge much mucous liquid, and sometimes blood.

*Prescriptions.* Sumatur primo mane, p. o. f. Ol. Ricini, fʒj. vel ij.—Aquæ puræ, f℥vj.; Potass. Nitrat. ʒss.; Tinct. Digital. m. xx.; Liquor Potass., Tinct. Iodini, aa fʒss.; Tinct. Hyoscyam. fʒj. Sumatur f℥j. ter quotidie.—Pulv. Glycyrrhiz. gr. xij.; Acet. Morphiæ, gr. ij.; Syrupi, q. s. Fiant pilulæ vj. quarum sumatur una, omni vesp. horâ somni.

15. The seton produces some discharge; but the legs are again œdematous, the pulse is weaker, and the boy seems evidently to be sinking.

*Prescriptions.* Rep. med. præter Tinct. Digital.

---

From this period he gradually declined, and died on the

20th of April, about eight o'clock in the morning. During the two or three last days of his life, he seemed to be conscious that his end was approaching. His principal symptoms at this time indicated congestion and oppression of the lungs; namely, troublesome cough, with bloody expectoration, and increased difficulty of breathing, and of lying down. He also complained much of an oppressive sense of heat about the head, requiring free ventilation; and, on the day before his death, with a view to obtain relief, cut off a good deal of his hair with his own hands.

*Post-mortem Appearances.* On inspecting the body early in the morning of April 22, the following appearances were observed.

*General Conditions.* Released from the contracted posture induced by the disease during life, the body appeared tall, and well made, and, although reduced, was not emaciated. There was no œdema, but the posterior surface was stained of a violet colour, extending to the sides of the neck. The face was rather livid and bloated, with bloody coagula adhering to the lips and nostrils, but its expression was natural. The limbs were flexible. The skin was in general pale, thin, and semitransparent, but sufficiently firm. The muscles were wasted, but of a good colour. There was little blood in the body, except in the heart and lungs; and scarcely any fat, except a slight layer under the skin, and that attached to the colon. Putrescence, although not commenced, seemed to be approaching, and was perhaps promoted by the influence of alimentary liquid in the stomach, which had become sour, and partially escaped from the mouth.

The head and spine were not examined.

*Thorax.* The left side of the chest was more prominent than the right, and yielded a duller sound on percussion. Ths muscles about the clavicles were thick and strong, owing, probably, to their increased action duiing life. The cartilages of the ribs were firmer than usual. The mediastinum was short and diffuse.

The heart was greatly enlarged, so as to occupy all the front of the chest from the top to the bottom. The left lung was, in consequence, pushed out of sight, and only a small portion of the anterior edge of the right lung, towards its lower end, was visible on removing the sternum. The pericardium adhered by numerous short filaments to the pleura lining the ribs and diaphragm, and its two layers were closely united into a thick dense membrane, of a dark grey colour, which was not easily penetrated by the scalpel, and could only be dissevered by laborious dissection. The heart was uniformly hypertrophied, both in length and in breadth, and of about four times its natural size. It contained a large quantity of blood, partly in the state of dark-red coagulum, very soft and almost liquid, and partly in that of firm yellow polypoid masses, chiefly occupying the auricles, especially the left auricle, and the arterial trunks. The lining membrane of the auricles was somewhat thickened and blanched, and the fossa ovalis was indistinct. That of the right auricle was roughened on the side towards the septum by a cluster of slender parallel ridges, apparently occasioned by an interstitial deposit of lymph. The auriculæ propriæ were small, but strong; the musculi pectinati plump and loose. The cavity of the right ventricle was narrow and oblong, extending chiefly in the direction of the pulmonary artery; that of the left ventricle was capacious. The walls of both were thick, but, owing to the regular and uniform character of the hypertrophy, not excessively so. In the left ventricle, the carneæ columnæ exhibited in some places a sort of internal layer, partially detached from the external one. The cardiac valves were slightly tumid and opaque, as were also those of the aorta at their bases, so as to resemble the valves of an old heart, but without occasioning any impediment to their function. With the exception of the inferior vena cava, the vascular trunks, and especially the pulmonary veins, were rather small, and appeared as if they had been subjected to the constricting influence of the inflamed pericardium.

The right lung was attached by several narrow bands of adhesion to the ribs and diaphragm, but less firmly to the former. The lower portion of the costal pleura was deeply reddened, and there was a small quantity of bloody serum in the pleural sac. The lung itself was perfectly sound, free from tubercles, hepatization, and œdema, but so much congested and condensed as rather to float under water than upon it. Its substance was red and firm, but quite permeable to air. In the upper portion, the pulmonary veins were obviously dilated, and the pleura was spotted here and there with small white patches, as if from incipient emphysema. The lower lobe was more especially congested, and of a deep cherry colour, with few or no pores. The bronchial membrane was reddened, and contained a little frothy mucus. The glands were rather soft and pale, and considerably enlarged. The condition of the left lung was nearly similar to that of the right one, but it was more crepitant, and at the same time partially œdematous. Between the pleural surfaces there was little adhesion, and no effusion. The lower lobe seemed to have been pushed upwards by the enlarged heart, and to have been gradually reduced by absorption.

*Abdomen.* The peritoneal sac was semitransparent, and nearly free from extravasated liquid. The omentum was somewhat retracted towards the left side. The stomach was large and thin, the pylorus slender and open. The outer surface of this organ was rather blanched, the inner one slightly corrugated. The upper part of the small intestine was white and thick, as if sodden; the lower portion thin and nearly transparent, as if verging to atrophy. Its contents were chiefly gaseous. With the exception of the cœcum, the large intestine was rather contracted, but was lined throughout by tenacious yellow fæces. Some of the mesenteric glands were a little red and tumid. The colon was studded with a few appendiculæ epiploicæ, but there was no other fat in the abdomen.

The liver was large and heavy, and attached to the adjacent parts by several bridles of adhesion. Its peritoneal coat was of a dark colour: its substance was dense, and slightly hypertrophied, but nearly natural. Some portions were rather pale, but without mottling. The left lobe was of considerable bulk, as was also the lobulus Spigelii, which presented a sort of caudal projection. The vena portæ was capacious, but not over-distended with blood. The gallbladder, which was rather thin and spacious, was half filled with pale dilute bile. The spleen was flat and oblong, and of nearly double its natural size: internally it was of a pink red colour, and of firmer consistence than usual, with a few membranous interstices, or septula. The pancreas was large and loose, and the cellular membrane interspersed between its lobules was red and injected. The kidneys were very firm and fleshy, and of full size: their cortical substance was thick, of a deep red colour, and in some places bloody on section, but perfectly sound. The bladder contained several ounces of transparent high-coloured urine.

Remarks. This was evidently a case of simple hypertrophy of the heart, resulting from chronic carditis, excited during convalescence from continued fever, by repeated chills of the legs and feet. The existence of inflammation was sufficiently proved by the adhesion of the pericardium to the adjacent organs, by the intimate consolidation of its two layers, the developement of the muscular substance of the heart, and the incipient thickening of its lining membrane, amounting in some places to an obvious interstitial deposit. When carditis is occasioned by refrigeration of external parts, rheumatism of some of the larger joints or muscles usually intervenes; but, in this instance, the effect seems to have been produced directly, without any intermediate condition.

The proximate symptoms of the complaint were very plainly marked,—namely, pain and palpitation in the region of the heart, the action of which was too strong, and too widely diffused; together with dry cough, hurried respiration, a weak respiratory murmur in the lower part of the chest, and, ultimately, bloody expectoration. The more remote symptoms were equally conspicuous, depending chiefly, as it seemed, on impeded return of blood from various organs, with consequent congestion, and injury of function. In the head, this condition was denoted by turgescence of the external vessels, profuse sweating, and occasional epistaxis, or mucous discharge from the nostrils; also, by irritability of temper, disposition to pick the nose, and other parts of the body, and by disturbed sleep, attended with moaning, or grinding of the teeth. In the liver, it was marked by fulness of the hypochondria, and at times by severe pain in the right side; in the alimentary canal, by impatience of repletion, and by frequent evacuations; in the kidneys, by red and turbid urine; and, in the lower extremities, by œdema, and articular pains.

The diagnosis thus clearly resulting from the influence of the hypertrophied heart on the lungs, liver, and stomach, was rendered still more distinct by the acoustic signs arising from auscultation and percussion, as likewise by the altered form of the chest. The agency of certain passions in producing temporary distention of the heart, was well displayed, on several occasions, by a sudden aggravation of the cough and dyspnœa, when the patient was vexed or irritated; and by their equally rapid subsidence, as soon as the mind was tranquillized.

The cadaveric appearances so exactly corresponded with the symptoms, the pathognomic signs, and the general character of the complaint, that they had been pretty accurately predicted during life. Independently of the usual results of chronic inflammation in the heart and its appendages, as already noticed, the lungs and the solid viscera of the abdomen were all more or less congested and condensed, the alimentary canal alone being more free from alteration than is commonly observed. Among other useful lessons furnished by this and similar cases, they serve to prove that irregular and obstructed circulation, with all its manifold consequences, may arise, without structural change, from simple disordered action of the heart and blood-vessels; and they show the importance of a due degree and proportion of vital action in the various parts of complex organs, the loss of which may readily give rise to inflammation or congestion.

The prognosis of hypertrophy of the heart, is, perhaps, generally unfavourable; but, in young persons, the natural resources of the system are so great, that, unless excessive derangement has taken place, they often survive when older patients would perish; and, by the gradual changes attendant on growth and nutrition, they are sometimes entirely relieved, after two or three years, from all traces of their original disease.

The principal object of the treatment in such cases is evidently to diminish the bulk and action of the overgrown heart, without unduly weakening the general frame. In the present instance the practice was probably too debilitating, and more adapted to the primary inflammation, than to its advanced stage, or ultimate result. The violent action and increased volume of the heart naturally suggest the use of lowering measures; but, unless much caution is observed, these may sometimes be carried further than the system will admit. The opposite extreme of an over-stimulating treatment is equally injurious; and, hence, an intermediate plan is manifestly indicated, consisting in a tranquil mode of life, aided by country air, passive exercise, a middle diet, and a due attention to the excretions. Active remedies should, perhaps, for the most part be avoided, and the homœopathic, or expectant, practice preferred; but, if any exception is allowed, iodine in moderate doses, and with suitable auxiliaries, seems to be the most appropriate medicine, possessing, as it does, when properly managed, the invaluable property of exciting absorption, and reducing morbid bulk, without occasioning proportional debility.

